# An Actinobacterial Isolate, *Streptomyces sp*. YX44, Produces Broad-Spectrum Antibiotics That Strongly Inhibit *Staphylococcus aureus*

**DOI:** 10.3390/microorganisms9030630

**Published:** 2021-03-18

**Authors:** Tien-Lin Chang, Tzu-Wen Huang, Ying-Xuan Wang, Chang-Pan Liu, Ralph Kirby, Chien-Ming Chu, Chih-Hung Huang

**Affiliations:** 1 Graduate Institution of Engineering Technology-Doctoral Department, National Taipei University of Technology, Taipei 10608, Taiwan; shinya.chang@gmail.com (T.-L.C.); jimmychu62@gmail.com (C.-M.C.); 2 Institute of Biochemical and Biomedical Engineering, Department of Chemical Engineering and Biotechnology, National Taipei University of Technology, Taipei 10608, Taiwan; anita800826@gmail.com; 3 Department of Microbiology and Immunology, School of Medicine, College of Medicine, Taipei Medical University, Taipei 11031, Taiwan; tw.huang@tmu.edu.tw; 4 Division of Infectious Diseases, Department of Medicine, MacKay Memorial Hospital, Taipei 10491, Taiwan; JOELIU5929@hotmail.com.tw; 5 Department of Life Sciences and Institute of Genome Sciences, School of Life Sciences, National Yang Ming Chiao Tung University, Taipei 11221, Taiwan; rkirby@ym.edu.tw; 6 BioMedical Development Center, MacKay Memorial Hospital, New Taipei City 25160, Taiwan

**Keywords:** *Streptomyces*, antibiotic, pH stability, hydrophilic, multidrug-resistant *Staphylococcus aureus*

## Abstract

The need for new antibiotics is increasing due to their overuse, and antibiotic resistance has become one of the major threats worldwide to public health, food safety, and clinical treatment. In this study, we describe an actinobacterial isolate, YX44, which belongs to the genus *Streptomyces*. This *Streptomyces* was isolated from a drinking pipe located in Osaka, Japan, and has the ability to inhibit Gram-positive bacteria, Gram-negative bacteria, and various fungi. YX44 fermentation broth shows strong activity against *Escherichia coli* and *Staphylococcus aureus*, as well as also inhibiting clinical isolates of multidrug-resistant *Staphylococcus aureus*. The YX44 antibacterial substances in the broth are relatively heat-stable, show high stability from the pH range 1 to 11, and have good solubility in both organic and non-organic solvents. Size-exclusion chromatography revealed that the YX44 antibacterial compounds are less than 1000 Da in size. LC-MS was able to identify three possible candidate molecules with molecular weights of 308, 365, 460, and 653 g/mol; none of these sizes correspond to any well-known antibiotics. Our results show that *Streptomyces sp.* YX44 seems to produce a number of novel antibiotics with high pH stability and good solubility that have significant activity against *S. aureus,* including multidrug-resistant strains.

## 1. Introduction

*Streptomyces*, a Gram-positive bacterial genus, grows in a wide range of environments. The genus is made up of aerobic spore-forming actinobacteria that have been classified for many years based on morphological, chemotaxonomic, and physiological characteristics; this has recently been supplemented by molecular approaches [1]. More than 500 *Streptomyces* species have been described [2] and the *Streptomyces* is the largest bacterial genus [3]. One of the major characteristics of *Streptomyces* is their complex secondary metabolism [4]; their secondary metabolites have been retained as a major source of antibiotics [5]. They are the most prolific producers of bioactive compounds and are known to produce over two-thirds of clinically-used antibiotics of natural origin [6]. However, recently, the resistance of pathogens to antibiotics has become a growing global public health concern. Multidrug-resistant *Staphylococcus aureus* is an example of the emergence of multiple drug resistance among Gram-positive bacterial pathogens. Such bacteria have become a common source of infection in hospitals. In European countries, multidrug-resistant *S. aureus* makes up about 20% of *S. aureus* isolates from the clinical environment [7]. Similarly, various Gram-negative pathogens have also become resistant to various β-lactams, aminoglycosides, and fluoroquinolones. Both Gram-positive and Gram-negative resistant strains are a threat to therapeutic choices when treating patients [8]. Therefore, the discovery and exploitation of new antimicrobials are urgently needed to combat these emerging drug-resistant pathogens.

In order to identify novel antibiotics, a number of different approaches have been used. Studies have revealed that secondary metabolic clusters exhibit strain-level diversity [9], which means a given novel strain often is capable of producing a novel secondary metabolite that is capable of being developed into clinically useful antibiotics. In this context, scientists have expanded their search range from the initial source of *Streptomyces*, namely the soil on land, to the oceans [10] and thence to many more diverse locations in order to isolate new strains of antibiotic-producing bacteria. Furthermore, technological improvements have allowed genome mining [11] and the activation of novel silent gene clusters [12] in strains, and this has helped to identify a number of novel compounds that have been worthy of further study.

In the present study, we performed a straightforward screening for bacteria that produce novel antibiotics from water-enriched environments such as rivulets, ponds, and water pipes at various locations. A range of *Streptomyces*-like isolates were identified from water in a drinking water pipe; one strain was found to produce what seems to be a number of novel antibiotics. These compounds are hydrophilic with a wide pH tolerance and broad spectrum in terms of their target organisms. They can inhibit both Gram-positive bacteria and Gram-negative bacteria, including multidrug-resistant *S. aureus*. Thus, these antibiotics have potential and are worthy of further study that might allow them to be developed as candidate antibiotics.

## 2. Materials and Methods

### 2.1. Isolation of and Maintenance of the Organism

Water samples were obtained from a water pipe in Osaka, Japan. These samples were diluted appropriately, plated onto Yeast Malt (YM) agar [13] and incubated at 30 °C for five days. Next, individual isolated colonies were picked and streaked separately onto YM plates to give isolated colonies; in total more than a hundred isolates were obtained. These plates were cultured to allow investigation of each isolate’s potential as a candidate producer of antibiotics. Any candidate *Streptomyces* that showed inhibition of a range of test microorganisms was grown until fully sporulated and then, individually, the spores of each isolate were harvested into sterile 20% glycerol and stored at −80 °C.

### 2.2. DNA Amplification by PCR and Determination of 16S rRNA Gene Sequence

The 16S rRNA sequence of the candidate isolate was amplified by PCR (Table 1) [14], sequenced, and subjected to BLAST against the NCBI GenBank database to identify a range of closely related strains of *Streptomyces*. Phylogenetic trees of the 16S rRNA sequence of the candidate strain and the related actinomycetes were constructed using Molecular Evolutionary Genetics Analysis (MEGA) software version X.

### 2.3. Detection of Antimicrobial Activity of YX44

Based on their 16S RNA molecular characterization, the antimicrobial spectra of the potentially novel *Streptomyces* strains were assessed. These strains were evaluated to determine their antimicrobial activity. This was initially carried out by cross-streaking the new isolates with various test strains on YM plates and incubating at 30 °C. The ability to inhibit the various test strains was recorded and compared across the isolated strains. One strain, YX44, stood out when tested against *Pseudomonas aeruginosa* ATCC 11633, *Escherichia coli* DH5α, *Staphylococcus aureus* ATCC 12145, *Candida albicans* ATCC 10231, *Saccharomyces cerevisiae* ATCC 9763, and *Aspergillus niger* ATCC 16404.

### 2.4. Inhibition of the Test Strains by YX44 Fermentation Broth

Liquid fermentation was carried out on the standout strain YX44 using different culture conditions. An inoculum of 200 µg of YX44 was added into 100 mL of YM medium in a 300 mL flask, and incubated at 30 °C on a rotary incubator at 200 rpm for 3 days. The YX44 fermentation broth, after settling, was freeze-dried and this was followed by resuspension to give a 100× concentrated solution. Next, 1.5 µL of concentrated solution was applied to a sterile paper disk placed on a YM plate. The YM plates had been previously spread with the various tested microorganisms as detailed above. The plates were cultured at 30 °C for 2 days. The inhibition zones were measured in order to evaluate whether the liquid fermentation had the same antimicrobial profile as the solid-state culture of YX44.

Inhibition testing against clinical isolates of multidrug-resistant pathogen strains, including multidrug-resistant *S. aureus*, was carried out at Mackay Memorial Hospital, Taipei, Taiwan, using 10× concentrated liquid fermentation broth. Three clinical isolate strains were used for this test. The antibiotic-resistance profiles of these strains and the inhibition effects of the fermentation broths were noted.

### 2.5. Characterization of the YX44 Antibacterial Products

To understand the basic characters of the antibacterial substances produced by YX44, their pH stability, temperature stability, solubility, and molecular size were investigated. For the pH stability test, freeze-dried YX44 fermentation broth was adjusted to either pH 1 or to pH 13 for 1 h, then neutralized to pH 7. To evaluate the potential effects of oral administration when treating a GI tract infection, the gastric environment was simulated by adjusted the YX44 fermentation broth to pH 2 and this was accompanied by incubation at 37 °C for 6 h, which was followed by neutralization to pH 7 [15]. Thermal stability was investigated by holding the YX44 fermentation at the following temperatures, 25 °C, 37 °C, 60 °C, and 100 °C, for various periods. The storability of the fermentation products was investigated by holding samples of the freeze-dried fermentation broth at 4 °C, room temperature, and 30 °C for 2 weeks. Finally, n-butanol and ethyl-acetate were used to determine if the active products in the vacuum-dried YX44 fermentation were soluble and remained active when extracted into these organic solvents. In all the above investigations, *S. aureus* was used to assess the antibacterial activity of the fermentation products present in the YX44 fermentation broth.

### 2.6. HPLC and LC-MS/MS Analysis

In order to separate the YX44 antibacterial substances based on molecular weight, 200 µL of 10× concentrated YX44 fermentation broth was loaded onto a size exclusion chromatography (SEC) column (SB-802.5-4E, 4.6 mm × 250 mm, exclusion limit: 10,000 Da, SHODEX, Tokyo, Japan). The mobile phase consisted of two solvents, A (ddH_2_O) and B (methanol). The elution mode was programmed as follows: 0% to 100% B from 0 min to 30 min, then 100% to 0% B from 30 min to 60 min. The flow rate of the mobile phase was set at 200 µL/min and fractions were collected every minute. UV detection was carried out at 280 nm using a Photodiode Array (PDA) detector and the readings were recorded. Each fraction was assessed for antimicrobial activity against *S. aureus*.

### 2.7. ESI-MS

The ESI(+)MS experiments were carried out using a LTQ-Orbitrap hybrid tandem mass spectrometer (ThermoFisher, Waltham, Massachusetts, USA) that was equipped with an electrospray ionization (ESI) source operating in positive ion mode and set up in line with an Agilent 1200 HPLC system. The HPLC column was an Agilent mRP-C18 High-Recovery Protein Column (length: 100 mm; internal diameter: 0.5 mm; bead size: 5 μm). The mobile phase consisted of (A) 0.1% formic acid in water and (B) 0.1% formic acid in acetonitrile. The parameters of the ESI(+) consisted of 4.0 kV ion spray voltage, 200 °C capillary temperature, and sheath gas flow rate at 3–5 arbitrary unit. Mass spectra were collected over the mass range of *m*/*z* 50–2000 at a resolving power of 30,000. After collection, the data were analyzed using Xcalibur software (ThermoFisher, Waltham, MA, USA). The molecular masses of the potential antibiotic molecules detected by ESI-MS were compared with those of known natural antibiotics.

## 3. Results and Discussion

### 3.1. A New Actinobacterial Species, Streptomyces sp. YX44, Has Broad-Spectrum Antibacterial Activity in Solid Culture

**Figure 1:**
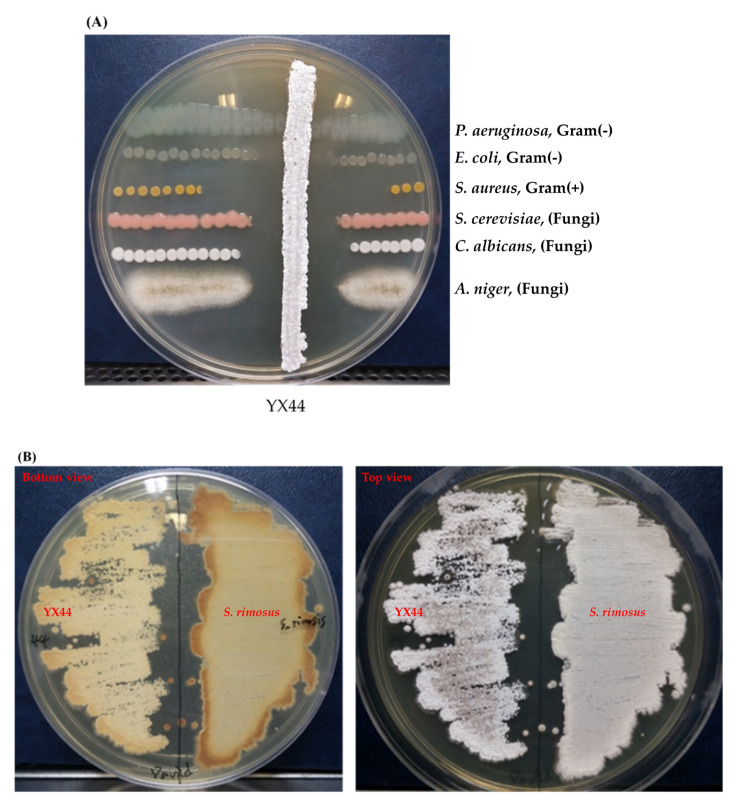

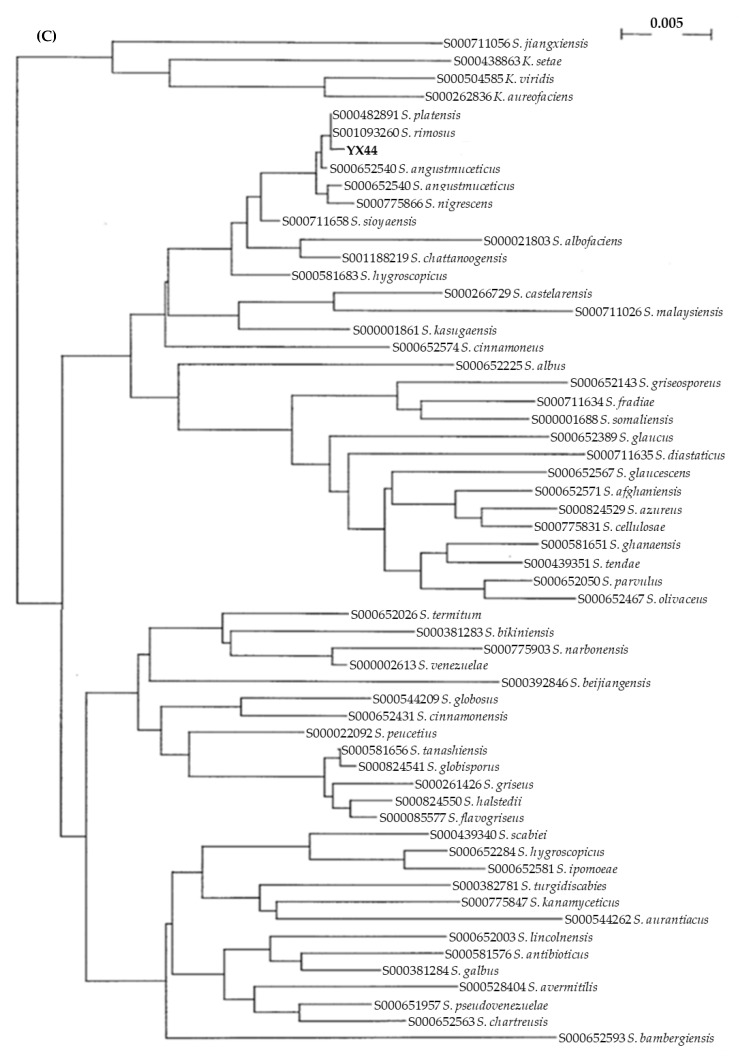
(**A**) Antimicrobial activity testing of YX44, which was inoculated onto YM agar for 7 days. An amount of 1 µL of bacterial culture containing 10^5^ testing pathogens were applied at both sides of YX44 to estimate the antimicrobial ability and spectrum. (**B**) Solid culture of YX44 (left) and *S. rimosus* (right) using YM plate. YX44 substrate mycelium is grey in color and has a smooth surface, while the aerial mycelium of *S. rimosus* is white in color and has a much rougher surface due to spore formation. (**C**) Maximum likelihood phylogenetic tree based on 53 *Streptomyces* 16S rRNA gene sequences and two outgroup 16S rRNA gene sequences. This shows the relationship of strain YX44 against other members of the genus *Streptomyces*. Initial tree(s) for the heuristic search were obtained automatically by applying the neighbor-joining and BioNJ algorithms to create a matrix of pairwise distances that were estimated using the maximum composite likelihood (MCL) approach. The topology of the final tree was then selected using superior log-likelihood values. The tree is drawn to scale, with branch lengths indicating the number of substitutions per site between species. This analysis involved 58 nucleotide sequences. All positions containing gaps and missing data were eliminated (complete deletion option). There was a total of 480 positions in the final dataset. Evolutionary analyses were conducted using MEGA X.

Of the 116 isolated Actinomycetes-like samples obtained from the water pipe that were tested, sample No. 44 (named as YX44) was a strain that showed significant inhibition of a number of microbial test strains. The pathogen strains chosen for testing were Gram-positive (*S. aureus* ATCC 12145), Gram-negative (*P. aeruginosa* ATCC 11633 and *E. coli* DH5α), and the fungi (*S. cerevisiae* ATCC 9763, *C. albicans* ATCC 10231, and *A. niger* ATCC 16404). The test results showed that YX44 inhibited all of these pathogen strains (Figure 1A), with the strongest activity being against *S. aureus* and the weakest activity being against *P. aeruginosa*. These findings suggest that YX44 produces antimicrobial molecules that are able to inhibit a wide range of microorganisms, including Gram-positive bacteria, Gram-negative bacteria, and fungi. 

**Figure 2:**
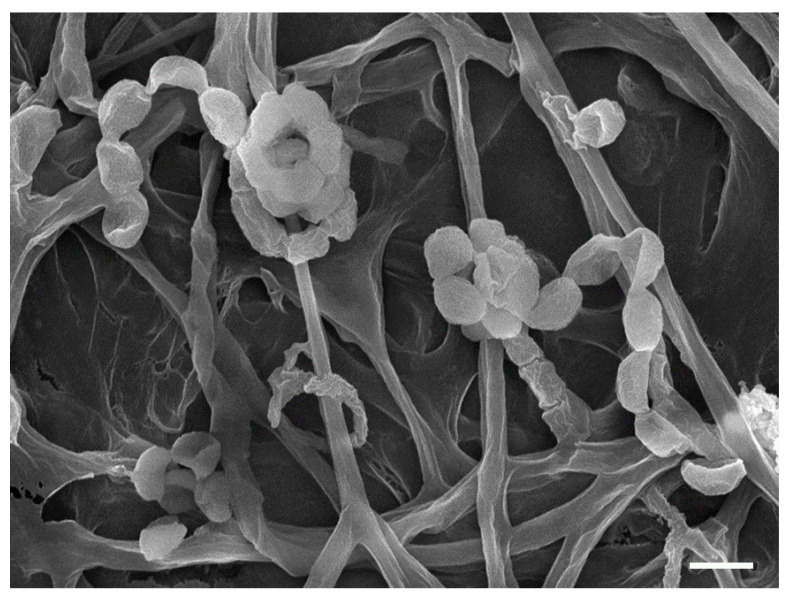
Scanning electron micrograph of YX 44 grown on soya flour mannitol (SFM) agar for 2 weeks at 30 °C; bar 1 µm.

YX44 showed sporulation under solid culture conditions similar to those used for other *Streptomyces*; this suggested that YX44 might be a member of the Actinomycetes and is probably a *Streptomyces* (Figure 1B). A pairwise comparison with various 16S rRNA gene sequences from the NCBI GenBank database was carried out and YX44 was found to have the highest 16S rRNA identities with *Streptomyces rimosus*, *Streptomyces platensis*, and *Streptomyces angustmyceticus*. This relationship was supported by a phylogenetic analysis using *Streptacidiphilus jiangxiensis*, *Kitasatospora viridis*, *Kitasatospora aureofaciens,* and *Kitasatospora setae* as outgroups (Figure 1C). These closely related *Streptomyces* strains are known to produce a range of useful antibiotics. In addition to the above findings indicating the YM44 is a *Streptomyces*, micromorphological studies of the strain using SEM showed coil-shaped spore chains, bowl-like spores, and a rectiflexibilis spore morphology (Figure 2), all of which are characteristic of a member of the *Streptomyces*.

### 3.2. Liquid Culture of YX44 Shows an Altered Antibiotic Profile

**Figure 3:**
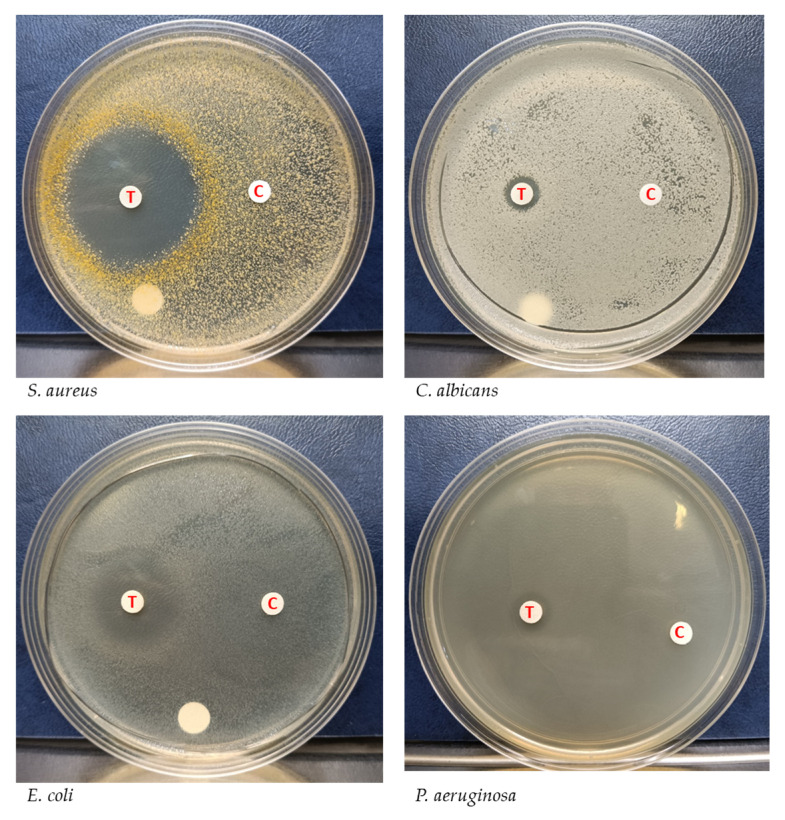
Inhibition of different microorganisms by *Streptomyces sp.* YX44 fermentation broth. Four pathogens, including *S. aureus*, *P. aeruginosa*, *E. coli*, and *C. albicans,* were used, 1.5 µL of the 100× concentrated fermentation broth was loaded onto each paper disk. T: *Streptomyces sp*. YX44 fermentation supernatant, C: YM medium as control.

In order to stably produce YX44’s antimicrobial substances for further analysis, liquid culture was carried out and the supernatant was used for further inhibition studies. *S. aureus*, *P. aeruginosa*, *E. coli*, and *C. albicans* were used to test the inhibition activity of the YX44 fermentation broth (Figure 3). YX44 fermentation broth produced significant inhibition of *E. coli*, while, on the other hand, inhibition of *C. albicans* was almost completely lost. The difference in inhibition profiles between solid-state culture and liquid culture suggests that YX44 may produce a number of distinct antibiotics that are able to target various microorganisms, and the change in the method of culture has altered the expression patterns of the secondary metabolic pathways within YX44. Various approaches, such as changing the bacteria-to-medium ratio, altering the culture period, and culturing YX44 with sterile pathogen supernatant were carried out in attempts to reproduce the broad inhibition activity against fungi. Unfortunately, none of the culture conditions used were able to produce the bioactive compound(s) that inhibited the fungi. The key factor(s) that would allow broad-spectrum antifungal activity in liquid culture remains unknown. One of the main differences between solid-state culture and liquid culture is that no sporulation of YX44 takes place in liquid culture and only substrate mycelium is present. It is very rare for a *Streptomyces* to sporulate in liquid culture and it is well known that this can have an effect on antibiotic production by *Streptomyces* [16,17].

Based on the strong inhibition of *S. aureus* in the presence of YX44 fermentation broth, the same broth was tested against three clinical multidrug-resistant strains of *S. aureus*. The YX44 antibacterial products in the broth were able to inhibit *S. aureus* strains that were resistant to clindamycin, oxacillin, trimethoprim-sulfamethoxazole, and daptomycin (Table S1). The diameter of inhibition zones against the multidrug-resistant *S. aureus* strains were in the range of 10 mm to 15 mm when 30 µL of broth was used (Figure S1). This activity was between 26% and 40% of the activity against the original *S. aureus* strain. These antibiotic resistance pathways are distinctly different, which suggests that the antibacterial chemicals produced by YX44 might either work differently from the above antibiotics or are structurally different enough to avoid the mechanisms at work in these antibiotic-resistant strains.

### 3.3. Characterization of the Streptomyces sp. YX44 Antibacterial Products

**Figure 4:**
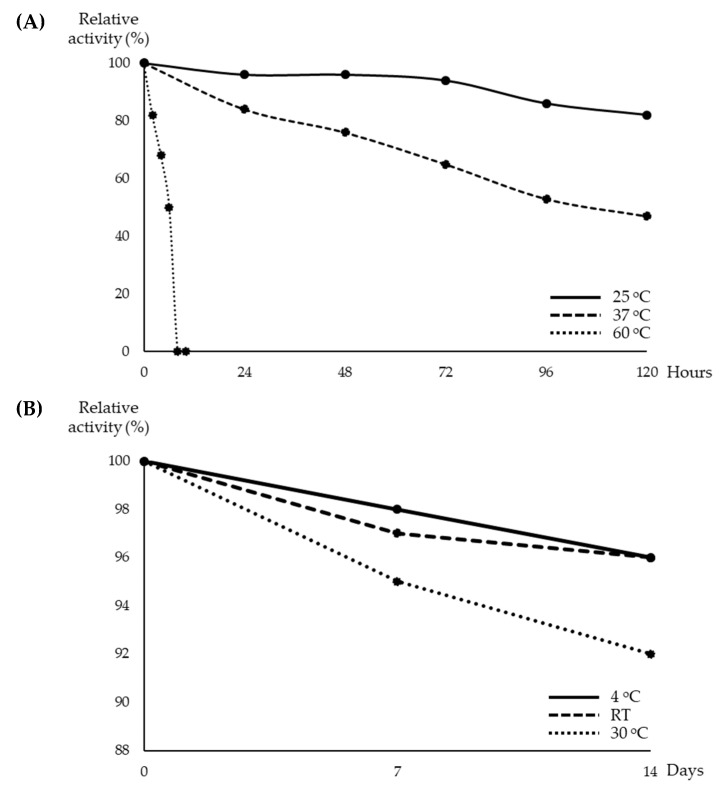
(**A**) Activity of YX44 fermentation broth stored under different conditions. (**B**) Activity of lyophilized YX44 fermentation broth stored under different conditions.

In order to further understand the characteristics of the *Streptomyces sp*. YX44 antibacterial products, the basic physical characters of these chemicals were investigated. The temperature stability test showed that the YX44 antibacterial substances were not stable at high temperatures. Inhibition activity was found to decline as the storage temperature was increased. At 60 °C, the YX44 antibacterial substances became inactive after 8 h (Figure 4A) while the broth completely lost its antibacterial activity at 100 °C within 2 min. When stored at lower temperatures, activity was more stable. At 25 °C, activity had dropped to around 82% after five days of storage (Figure 4A), while at 4 °C, activity was 98% after seven days (Figure 4B). Storability increased significantly when the YX44 fermentation broth was freeze-dried. There was only a loss of 4% to 8% of activity when freeze-dried YX44 fermentation broth was stored at 4 °C, room temperature, and 30 °C for two weeks (Figure 4B).

**Figure 5:**
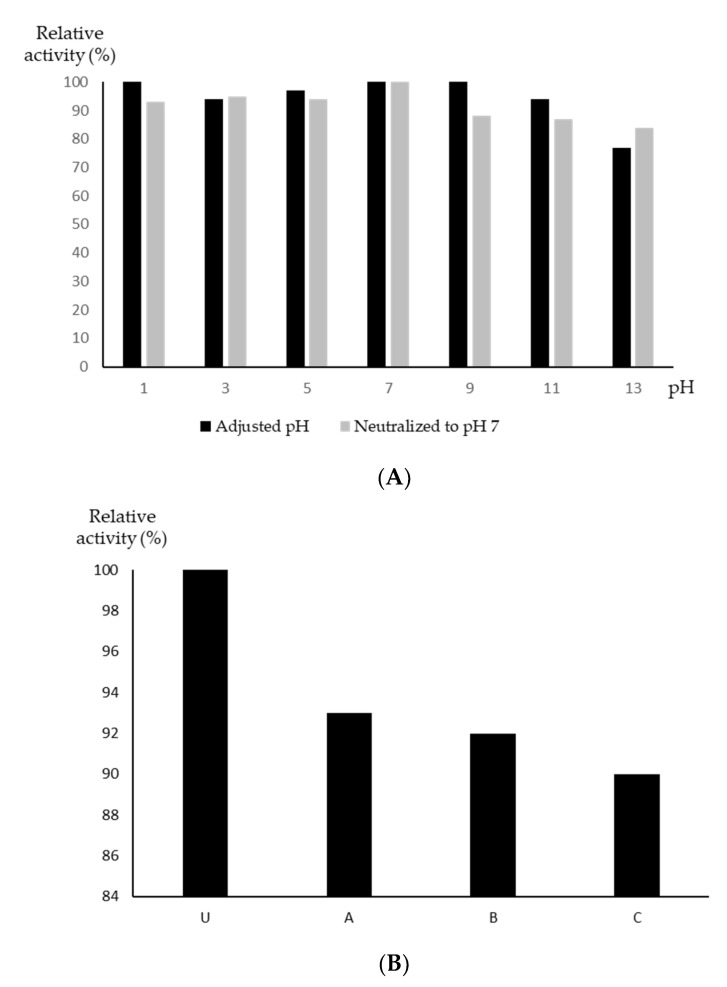
(**A**) pH stability test of *Streptomyces sp.* YX44 fermentation broth. (**B**) Stability of *Streptomyces sp.* YX44 fermentation broth under GI tract environment emulation. U: control; A: adjusted to pH 2; B: adjusted to pH 2, incubated at 37 °C for 6 h; C: adjusted to pH 2, incubated at 37 °C for 6 h, neutralized to pH 7.

While most antibiotics are most active at a relatively neutral pH, *S. aureus* in general, and multidrug-resistant *S. aureus* in particular, are capable of surviving at a much wider pH range, for example in the gastrointestinal (GI) tract [18] and on the skin. To our surprise, YX44 antibacterial products were found to be very stable over the range from pH 1 to pH 13, with inhibition only being reduced slightly to 76% at pH 13, with more than 90% of the activity remaining over the pH range 1 to 11. Furthermore, the increased concentration of salts that resulted from neutralization back to pH 7 had little effect on activity that had undergone a pH increase. When the pH of the broth was decreased, the loss of activity was more obvious (Figure 5A). However, when the pH pattern of oral administration via the stomach was simulated, the YX44 antibacterial products retained about 90% of their activity after 6 h of incubation at 37 °C (Figure 5B). Antibiotics usually have a pH range in which they are most stable, and once they move out of this range, they often become inactive quickly [19,20]. When treating *S. aureus* infections, a lack of stability under acidic conditions can limit the efficacy and choice of antibiotics [21]. The wide pH stability range of YX44 antibacterial products is a rare and important feature that should be helpful when targeting in vivo GI tract *S. aureus* infections [22,23]. Finally, the activity of YX44 fermentation broth remained unchanged after treatment with proteases, which suggests that the active compounds are not peptides (data not shown).

**Figure 6:**
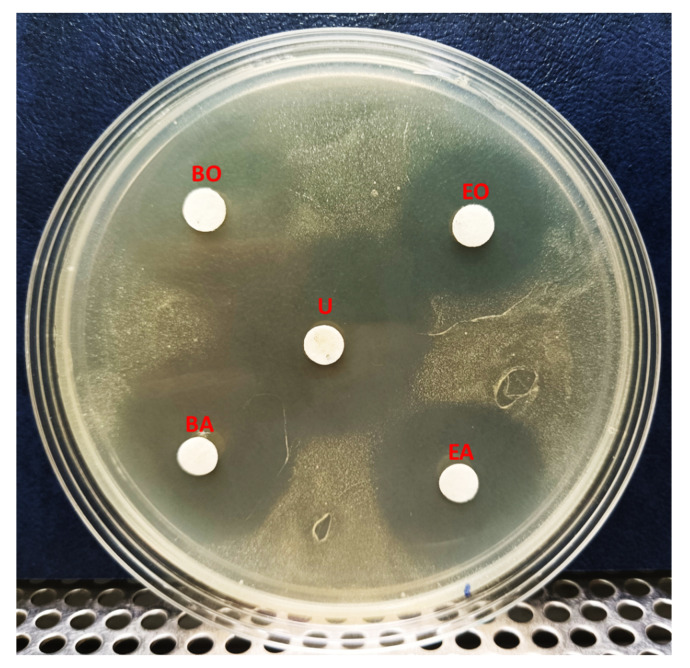
Solubility test of *Streptomyces sp.* YX44 fermentation broth. *S. aureus* was inoculated onto YM plate. U: untreated ferment supernatant, BO: n-Butanol organic phase, BA: n-Butanol aqueous phase, EO: Ethyl acetate organic phase, EA: Ethyl acetate aqueous phase.

We next investigated the solubility of YX44 antibacterial compounds by dissolving the freeze-dried YX44 fermentation broth in either n-butanol or ethyl-acetate. The active compounds from *Streptomyces sp*. YX44 were highly soluble in both of these solvents and retained their activity. However, when mixed with an equal volume of ddH_2_O, the YX44 antibacterial compounds quickly entered the water phase with few seconds of vortexing. The relative percentage of the inhibition zone associated with both aqueous phases was over 50%. It showed that they had higher solubility in water than the two organic solvents (Figure 6).

### 3.4. HPLC and LC-MS Analysis Revealed a Number of Candidates for the YX44 Antibacterial Compounds

**Figure 7:**
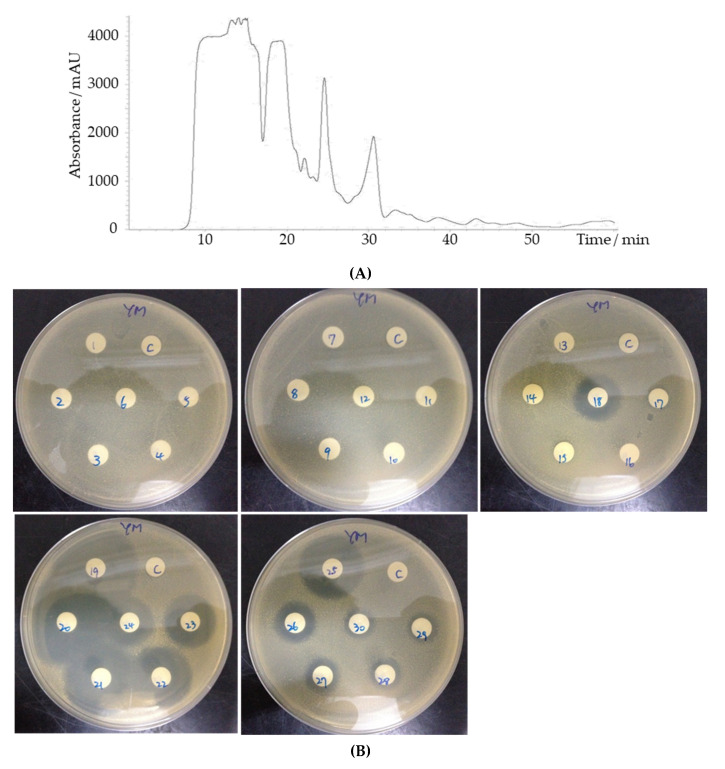
(**A**) HPLC separation of *Streptomyces sp.* YX44 fermentation broth by GFC. *S. aureus* was inoculated onto YM plate. The flow rate was 200 µL/min, fractions were collected every minute. (**B**) 1~30: fractions collected at different time points.

An initial purification of the YX44 antibacterial compounds showed that they were able to diffuse through a 2500 Da cutoff dialysis membrane easily, and that normal phase separation using a hydrophobic C18 column failed due to their high water solubility (data not shown). Based on these findings, separation was carried out by HPLC analysis using a gel filtration column (GFC). Antibacterial activity was present in a number of different fractions. An asymmetric inhibition activity pattern with multiple detected peaks was found for fractions 18 to 24, together with a sharp peak and strong inhibition activity at fraction 25 (Figure 7). This suggests that YX44 is producing a number of different antibacterial compounds in liquid culture. 

**Figure 8:**
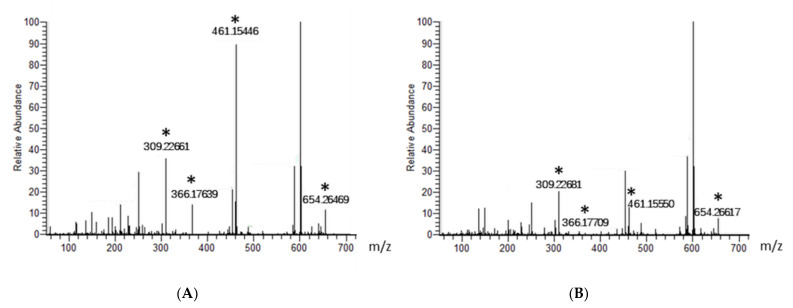
Identification of potential *Streptomyces sp.* YX44 antibiotic substances by LC-MS analysis. Fermentation broth (**A**) and a sample inactivated by heat treatment (**B**) were used as control and had been screened, respectively. Four major peaks shown significant changes between sample and control were labeled with asterisks.

In addition, the elution pattern indicated that all of these potential antibacterial compounds were smaller than 1000 Da in size, and this was confirmed by dialysis using membranes with different pore sizes and cut-off ranges (data not shown). Next, we subjected the YX44 fermentation broth to LC-MS analysis. The samples were filtered through a 1000 Da membrane to reduce noise, and half the sample was then heat-inactivated at 100 °C for 2 min as a control. Four peaks were clearly present when the active sample was compared to the control samples, and these had molecular sizes of 308, 365, 460, and 653 g/mol after deducting an extra proton due to the positive charge in sample preparation (Figure 8). One well-known antibiotic with good activity against *S. aureus* that has a size similar to these molecules is clindamycin hydrochloride, at 461.439 g/mol [24]. However, the YX44 compounds are able to inhibit clindamycin-resistant *S. aureus* (Figure S1, Table S1), and clindamycin is known to be a semisynthetic antibiotic with a significant decrease in activity against *S. aureus* under acidic condition (pH < 6) [25], which is quite different from the good pH stability of YX44 active compounds. Another well-known antibiotic that has a molecular weight close to 460 is oxytetracycline, at 460.4. Given the close phylogenetic relationship between *Streptomyces sp*. YX44 and a number of oxytetracycline producers, namely *Streptomyces rimosus* and *Streptomyces platensis* (Figure 1C), oxytetracycline homologs might be a candidate for one of the four active compounds produced by YX44.

## 4. Conclusions

With the increasing threat to patients of antibiotic resistance, the need for novel antibiotics has become a very serious issue. Researchers have expanded their search to many different environments, including bat caves [26], underground lakes [27], and deserts [28], with the aim of finding new candidate antibiotics. In this study, we screened water samples from a drinking pipe and found a *Streptomyces*, YX44, that exhibited promising broad-spectrum inhibition activity against a number of different pathogens. YX44 fermentation broth shows significant antibacterial activity against *S. aureus* and also inhibits clinical multidrug-resistant strains from a hospital. The exceptional pH stability of the compounds produced by YX44 is a unique great feature that could be useful clinically because many common antibiotics are affected by pH changes, which can affect their clinical application [29,30]. While the active compounds being hydrophilic matches the nature habitat of YX44, the good solubility of the active compounds in both organic and non-organic solvents is rare and potentially useful. The HPLC and LC-MS results indicate that there seems to be more than one active compound produced by YX44 and there are no reports of antibiotics that have characteristics similar to the YX44 candidate molecules. Our phylogenetic analysis indicates that *Streptomyces sp*. YX44 is a novel *Streptomyces* that can produce one or more new antibiotics with significant activity against *S. aureus*, including multidrug-resistant strains of *S. aureus*, while also showing antibacterial good activity over a wide pH range.

## Figures and Tables

**Table 1:** Primer set used for the amplification of the *Streptomyces* specific region of *Streptomyces sp*. YX44 16S rRNA for species identification.

Primer	Sequences
16S rRNA R	AGTTTGATCCTGGCTCAGGA
16S rRNA F	ATTACCGCGGCTGCTGGCAC

## Supplementary Materials

The following are available online at https://www.mdpi.com/2076-2607/9/3/630/s1, Figure S1: Inhibition of various clinical multidrug-resistant S. aureus strains by Streptomyces sp. YX44 fermentation broth, Table S1: Antibiotic-resistant patterns of three clinical multidrug-resistant S. aureus strains.
