# Supplementary material for: An Actinobacterial Isolate, Streptomyces sp. YX44, Produces Broad-Spectrum Antibiotics That Strongly Inhibit Staphylococcus aureus
Source: Microorganisms. 2021 Mar 18;9(3):630. doi: 10.3390/microorganisms9030630 (PMC8002889; doi:10.3390/microorganisms9030630)
Supplement: Supplementary file 1 [file microorganisms-09-00630-s001.pdf]

microorganisms

Supplementary Material:

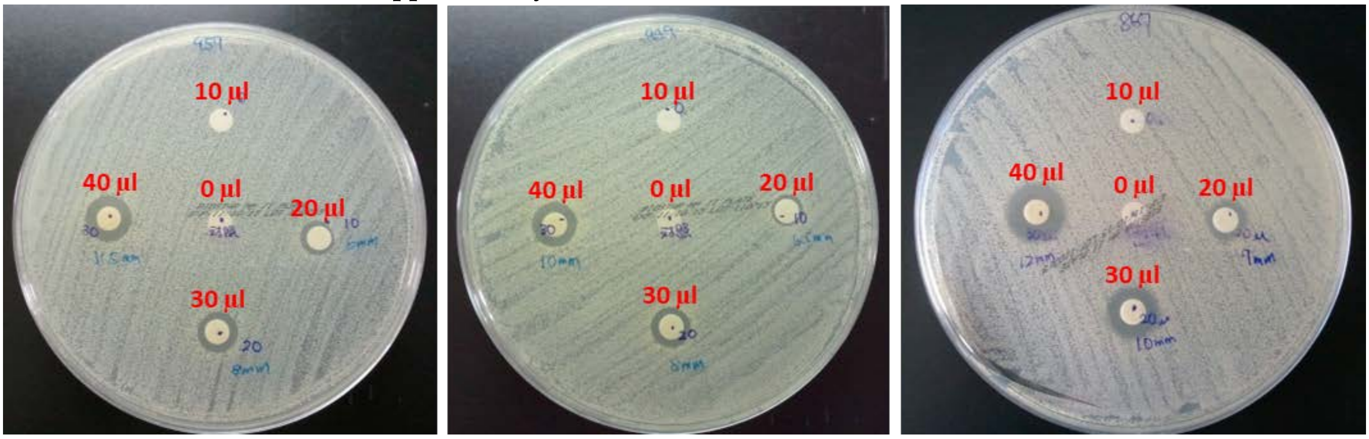

(A) (B) (C)

Figure S1. Inhibition of various clinical multidrug-resistant *S. aureus* strains by *Streptomyces sp.* YX44 fermentation broth. (A) multidrug-resistant *S. aureus* no. 957 (B) multidrug-resistant *S. aureus* no. 949 (C) multidrug-resistant *S. aureus* no. 867 were tested, the antibiotics resistant profile of tested strains are listed in Table S1.d

**Pathogenic Strain**	**Resistant antibiotics**
**Multidrug-resistant *S. aureus* no. 957**	**Clindamycin, Oxacillin, Trimethoprim-Sulfamethoxazole**
**Multidrug-resistant *S. aureus* no. 949**	**Clindamycin, Oxacillin, Trimethoprim-Sulfamethoxazole, Daptomycin**
**Multidrug-resistant *S. aureus* no. 867**	**Oxacillin**

Table S1. Antibiotic-resistant patterns of three clinical multidrug-resistant S. aureus strains.
